# The influence of societal individualism on a century of tobacco use: modelling the prevalence of smoking

**DOI:** 10.1186/s12889-015-2576-6

**Published:** 2015-12-22

**Authors:** John C. Lang, Daniel M. Abrams, Hans De Sterck

**Affiliations:** Department of Applied Mathematics, University of Waterloo, 200 University Avenue West, Waterloo, N2L 3G1 Canada; Department of Engineering Sciences and Applied Mathematics & Northwestern Institute on Complex Systems & Department of Physics and Astronomy, Northwestern University, 633 Clark Street, Evanston, 60208 USA

**Keywords:** Smoking, Individualism, Mathematical modelling, Social dynamics, Non-infectious diseases

## Abstract

**Background:**

Smoking of tobacco is estimated to have caused approximately six million deaths worldwide in 2014. Responding effectively to this epidemic requires a thorough understanding of how smoking behaviour is transmitted and modified.

**Methods:**

We present a new mathematical model of the social dynamics that cause cigarette smoking to spread in a population, incorporating aspects of individual and social utility. Model predictions are tested against two independent data sets spanning 25 countries: a newly compiled century-long composite data set on smoking prevalence, and Hofstede’s individualism/collectivism measure (IDV).

**Results:**

The general model prediction that more individualistic societies will show faster adoption and cessation of smoking is supported by the full 25 country smoking prevalence data set. Calibration of the model to the available smoking prevalence data is possible in a subset of 7 countries. Consistency of fitted model parameters with an additional, independent, data set further supports our model: the fitted value of the country-specific model parameter that determines the relative importance of social and individual factors in the decision of whether or not to smoke, is found to be significantly correlated with Hofstede’s IDV for the 25 countries in our data set.

**Conclusions:**

Our model in conjunction with extensive data on smoking prevalence provides evidence for the hypothesis that individualism/collectivism may have an important influence on the dynamics of smoking prevalence at the aggregate, population level. Significant implications for public health interventions are discussed.

**Electronic supplementary material:**

The online version of this article (doi:10.1186/s12889-015-2576-6) contains supplementary material, which is available to authorized users.

## Background

In the fifty years since the first report of the Surgeon General’s Advisory Committee on Smoking and Health [1] the smoking epidemic has been responsible for more than 20 million deaths in the United States alone [2, 3], and continues to be responsible for over 6 million deaths worldwide each year [4, 5]. The strong social component of the dynamics of smoking prevalence has been modelled mathematically [6–10], and examined statistically through analysis of social network data [11] and survey data [12–14]. However, whereas previous works tend to focus on the micro-level, in this paper we investigate how social aspects of smoking affect its prevalence at the societal level.

**Fig. 1:**
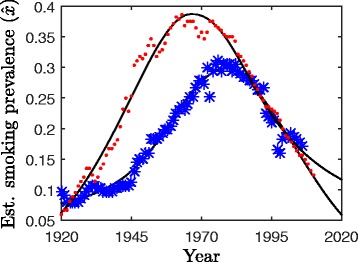
Estimated smoking prevalence in Sweden and the USA (1920-2020). Estimated smoking prevalence $\hat {x}$ versus time for the United States (dots) and Sweden (asterisks). The solid lines give the curves of best fit for Eq. (1)

Significant inter-country differences exist in smoking prevalence [15]. For example, Fig. 1 shows smoking prevalence estimates over most of the past century for Sweden and the USA, obtained from smoking prevalence surveys and cigarette consumption data (collectively referred to as tobacco use data, see Data subsection in Methods). In both countries, smoking prevalence increased rapidly starting from the early decades of the 20^th^ century and reached a peak in the 1960s–1980s era when the adverse health effects of smoking became widely known [1], after which smoking prevalence declined rapidly. However, there are conspicuous differences between the curves: the rate of smoking adoption and cessation before and after the peak is much greater in the US than in Sweden, and the peak in prevalence in the US occurs much earlier than in Sweden.

Considerable time and resources have been devoted to identifying the factors that contribute to smoking prevalence. Major factors include differences in beliefs about the harm of smoking [16], socio-economic status [17, 18], cost [19], regulation/tobacco control policies [20–22], and gender [23]. However, we note that these advances in the understanding of the factors contributing to smoking prevalence are based primarily on micro-level data, methods that inform general hypotheses, and non-mathematical descriptive models. Indeed, comprehensive and quantitative cross-national analyses of how all these factors affect smoking prevalence are rare [15]. Existing studies that compare national trends in smoking prevalence, as well as the factors that contribute to these trends, tend to take a descriptive [24, 25] and/or statistical [15] approach, and do not address the mechanism underlying the key decision of whether or not to smoke in a quantitative manner [26].

In this paper we present a new model for the social spreading of smoking. We aim to create and test a *tractable mathematical model*, that is, a model for qualitative dynamics from which insight (including causation) can be drawn. This differs from the statistically-driven approach often used in areas such as econometrics and medicine, where correlations may be uncovered and analyzed without formulating first-principle-based dynamic mathematical models. The statistical approach is difficult to apply here because the amount of available data on historical smoking dynamics is small. Our model-based approach has much in common with simple explanatory mathematical models that have been successful in, e.g., epidemiology and population dynamics.

Our model incorporates the concepts of *individual utility from smoking*, i.e. the utility an individual derives directly from the act of smoking (including awareness of health effects), and *social utility from smoking*, i.e. the utility an individual derives indirectly from smoking through social interactions with other smokers (peer influence and social inertia). Together these two quantities determine the *total utility from smoking*. Our model assumes that an individual’s decision to smoke is based on the desire to maximize total utility. By invoking this decision-making mechanism in a simple mathematical model, our approach differs from the approaches of the previous mathematical [7–10] and descriptive/statistical [15, 24–26] models. Whereas previous mathematical models generally require the calibration of many parameters (leading to difficulties in analysis, interpretation, and overfitting), we propose a simple approach based on principles of social psychology and sociology whose predictions can be directly compared to tobacco use data. Whereas previous descriptive and statistical models lack an underlying decision-making mechanism, we propose a model with a decision-making mechanism that is capable of incorporating factors previously identified as contributing to smoking prevalence. Specifically, we note that monetary cost, beliefs about the harm/health effects of smoking, and regulation/tobacco control policies are all implicitly accounted for in the concept of individual utility from smoking. Our simple model applies to the population level, focusing on major effects that may influence the temporal dynamics of smoking across societies. It proposes a mechanism for smoking adoption and cessation that hinges on the balance between individual and social utility (which both encompass other more fine-grained factors). Matching the model to real-world data reveals that the balance between social and individual utility indeed is an important factor in the temporal dynamics of smoking, differentiating between countries in a way that is consistent with known measures of societal individualism. This lends support to the compelling hypothesis that the balance between individual and social utility, which we will show to be related to societal individualism, is indeed an important society-level driver for the temporal dynamics of smoking prevalence. This is consistent with previous findings that the level of individualism/collectivism of a society may have fundamental implications for its biology [27, 28], as well as its behaviour [12, 29–32].

**Fig. 2:**
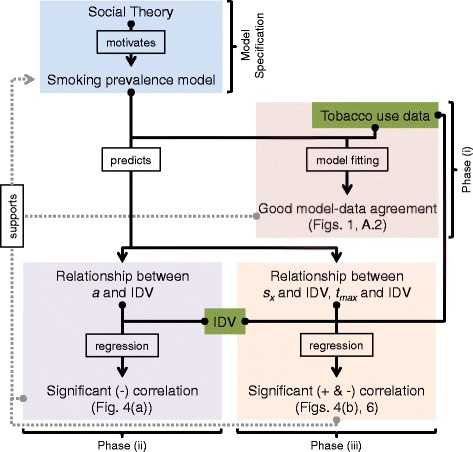
Model specification and testing of model predictions/interpretation in three phases. Schematic representation of the development and testing of the smoking prevalence model given in Eq. (1), see Results for detailed explanation. Logic flows from filled circles to arrow heads. Green rectangles enclose data sets used in this study. Model Specification (blue rectangle): social theory is used to motivate a mathematical model for the dynamics of smoking prevalence. Model predictions are tested in three phases. Phase (i) (red rectangle): the model is fitted to smoking prevalence estimates derived from tobacco use data (see Data subsection in Methods) resulting in good model-data agreement, see Fig. 1 and Fig. A.2 of Additional file 1. Phase (ii) (purple rectangle): the relative conformity parameter *a* from the model controls the relative contribution of social and individual utilities to total utility, and hence, is interpreted as reflecting the degree of individualism/collectivism in a society. This model prediction is tested by comparing country specific fitted values of *a* (calculated in Phase (i)) to Hofstede’s IDV, an already established measure of individualism/collectivism. Linear regression of *a* on IDV confirms that these two quantities are significantly (negatively) correlated, see Fig. 4(a). Phase(iii) (yellow rectangle): the relative conformity parameter *a* in Eq. (1) plays a central role in determining the rate of the increase/decrease in smoking prevalence. Our interpretation of *a* in terms of individualism/collectivism therefore predicts a relationship between the average rate of increase in smoking prevalence *s*_*x*_ (see Eq. (5)) and IDV, and between the year of peak smoking prevalence *t*_*max*_ and IDV. Linear regression of *s*_*x*_ on IDV confirms these two quantities are significantly (positively) correlated and linear regression of *t*_*max*_ on IDV confirms these two quantities are significantly (negatively) correlated, see Fig. 4(b) and Fig. 6, respectively. Results from Phases (i)-(iii) provide evidence in support of the model proposed in Model specification

The model we propose is explained in the Model specification subsection of the Methods section below. In the context of societal individualism/collectivism, the parameter in our model that controls the relative importance of individual versus social utility is interpreted as follows: the greater the relative contribution of individual utility to total utility (at the expense of social utility), the more individualistic the society is interpreted to be. Conversely, the greater the relative contribution of social utility to total utility (at the expense of individual utility), the more collectivistic the society is interpreted to be. As described in detail below (see Testing the model subsection in Methods), this allows us to test the model’s predictions against independently collected tobacco use and individualism/collectivism data sets in three separate phases, see Fig. 2. First, using tobacco use data we compile smoking prevalence estimates spanning the past century for seven countries belonging to the Organisation for Economic Co-operation and Development (OECD) and find good agreement between these estimates and the fitted model (Phase (i) in Model Testing, see Fig. 2). Second, the country-specific parameter in our model that controls the relative importance of individual versus social utility, i.e. the parameter that we interpret as the degree of societal individualism/collectivism (the *relative conformity parameter a*, see Model specification subsection in Methods), and that we fit to smoking prevalence estimates, is found to be significantly correlated to an established measure of societal individualism for each country (Hofstede’s IDV [33]), in agreement with the predictions of the model (Phase (ii) in Model Testing, see Fig. 2). Thirdly, given the predicted relationship between the relative conformity parameter *a* and Hofstede’s IDV (tested in Phase (ii)), and given the central role played by the relative conformity parameter *a* in our model, we are motivated to investigate directly the role that individualism (as measured by Hofstede’s IDV) plays in observed historical tobacco use data. Specifically, our model predicts that more individualistic societies will show faster adoption and cessation of smoking. We investigate this in historical tobacco use data, and find that IDV is significantly correlated to the average rate of increase in smoking prevalence (*s*_*x*_) in seven OECD countries for which historical smoking prevalence estimates are available, and that it is significantly correlated to the peak year of tobacco consumption (*t*_*max*_) for 25 countries in which tobacco consumption data are available, in agreement with model predictions (Phase (iii) in Fig. 2). These findings are interpreted according to our modelling framework, and provide evidence for the compelling hypothesis that individualism/collectivism has an important influence on the dynamics of smoking prevalence at the aggregate, population level.

## Methods

### Model specification

We begin formulating our model by observing that individuals derive utility from smoking via two mechanisms. First, they derive utility directly from the act of smoking (*individual utility*). Second, they derive utility from social interaction with other smokers (*social utility*). We note that social utility commonly manifests itself in the form of peer influence or peer pressure [34, 35]. We then proceed using a modelling framework that explicitly accounts for the effect of competition between individual and social utilities, and that was first applied to explore the temporal dynamics of language death and religious affiliation as binary choice problems [36, 37]. Specifically, we propose the model

(1) $$  \frac{dx}{dt} = b \left[(1-x) x^{a} u_{x} - x (1-x)^{a} (1-u_{x})\right],  $$

where *x*=*x*(*t*)∈ [ 0,1] is the fraction of smokers in the population (i.e., the prevalence) at time *t*, *u*_*x*_∈ [ 0,1] is the individual utility from smoking, and the constant *b*>0 determines the timescale of the equation. The interpretation of the positive term in Eq. (1), which models smoking adoption, is therefore that non-smokers 1−*x* take up smoking at a rate proportional to the *total utility* derived from smoking, *x*^*a*^*u*_*x*_, which is the weighted product of the individual utility from smoking *u*_*x*_ and the social utility from interactions with other smokers *x*, with weighting determined by the constant parameter *a*. Conversely, the interpretation of the negative term in Eq. (1), which models smoking cessation, follows analogously: smokers *x* cease smoking at a rate proportional to the total utility derived from non-smoking, (1−*x*)^*a*^(1−*u*_*x*_), which is the weighted product of the individual utility from non-smoking *u*_*y*_=1−*u*_*x*_ and the social utility from interactions with other non-smokers 1−*x*, where we have normalized individual utilities from smoking *u*_*x*_ and from non-smoking *u*_*y*_ such that *u*_*x*_+*u*_*y*_=1. Since societies with large *a* weigh changes in social utility more heavily than changes in individual utility when calculating total utility, we call *a* the *relative conformity parameter*. We therefore interpret societies with large *a* to be more collectivistic (or less individualistic) than societies with small *a*. In other words, since a society with *a*=1 weighs social and individual utility equally when calculating total utility, we expect strongly collectivistic societies to have *a*>1 and strongly individualistic societies to have *a*<1. We note that this modelling framework is conceptually consistent with the findings presented in [12]: that personal attitudes about smoking have a stronger influence on smoking behaviour in individualistic countries than in collectivistic countries. We also note that, although social utility follows from complex social interactions, we have made the simplifying assumption that the social utility of a group, e.g. of smokers, is proportional to the size of that group, e.g. the smoking prevalence *x*. This assumption has been shown to work well in previous works [36, 37].

Next, we observe that a combination of factors, including advances in our understanding of the health effects of smoking and public policy initiatives designed to curb smoking, have likely reduced individual utility from smoking (*u*_*x*_) over the past century. Thus, in a significant departure from previous work that treats individual utility as a constant [36, 37], we account for this decline in individual utility by using the cumulative number of scholarly articles on the health effects of smoking (*n*(*t*)) as a proxy for the reduction in individual utility over the past century. Since each additional article represents an increase in the public knowledge about the health effects of smoking, we assume that individual utility decreases with each additional article published. We also assume that public knowledge about the health effects of smoking becomes saturated after a large number of articles have been published. In other words, we assume that public knowledge about the health effects of smoking is subject to diminishing marginal returns from additional articles published, and hence, individual utility is subject to diminishing marginal losses from additional articles published. We apply these assumptions by following the principle of temporal discounting [38], i.e. we assume that each additional article published is discounted by the factor *δ*∈[ 0,1] so that for year *t*

(2) $$  u_{x}(t) = u_{\infty} + \delta^{n(t)} (u_{0} - u_{\infty}),  $$

where *u*_0_ and *u*_*∞*_ are the limiting individual utilities from smoking when there is no knowledge and perfect knowledge of the adverse effects of smoking, respectively. Here, *u*_0_, *u*_*∞*_ and *δ* are parameters to be fitted to observational data.

We remark that this approach leads to better fits between model output and observational data than alternatives that do not directly take into account the effect of increased scientific understanding of health effects. For example, using the discounting formula of Eq. (2) produces a better fit (significantly lower total error *E*_2_) than either constant utility *u*_*x*_(*t*)≡*u*_*x*_ or step-function utility

$$ u_{x}(t) = \left\{ \begin{array}{ll} u_{0} & \text{if}\,\, t<t^{*} \\ u_{\infty} & \text{if}\,\, t\geq t^{*} \end{array} \right., $$

where *t*^∗^ is a threshold parameter whose value is determined by the fitting procedure. Note that when *u*_0_>*u*_*∞*_ the step-function utility is consistent with the expectation that increasing knowledge of health effects has indeed influenced the individual utility from smoking over the past century.

### Data

We note that Eq. (1) subject to Eq. (2) requires the fitting of four parameters per country (*x*_0_=*x*(*t*_0_), *a*, *u*_0_, and *u*_*∞*_) and two parameters *b* and *δ* that we take equal for all countries in the data set (see Model Fitting in Methods). We determine these parameters by fitting them to estimated historical smoking prevalence data and proxy data on the health effects of smoking. We summarize the methods used to obtain these data below. Note: No human subjects participated in this study. No consent was necessary to obtain.

#### Tobacco use data: smoking prevalence and cigarette consumption data

We consider smoking prevalence *x*(*t*)∈ [ 0,1] for 24 OECD countries which we download from the OECD iLibrary online statistical database [39] in Excel format. We also consider manufactured cigarette consumption (in grams) per person per day *c*(*t*) for the same 24 OECD countries plus Romania (which is a non-OECD country) [40, 41]. When available, cigarette consumption data is downloaded directly from the International Smoking Statistics (Web Edition) website [40] in Excel format. Cigarette consumption data for countries not included in the International Smoking Statistics (Web Edition) are retrieved from the International Smoking Statistics (2^nd^ Ed.) [41] by manually transferring these entries into Excel. We refer to smoking prevalence and cigarette consumption data collectively as tobacco use data. We make these data available in CSV format in an additional file (see Additional file 2), which contains four columns: country number as it appears in Table A.1 of the Additional file 1, year (*t*), measurement (*x*(*t*) or *c*(*t*)), and type of measurement (0 indicates a smoking prevalence measurement, while 1 indicates a cigarette consumption measurement).

Unfortunately smoking prevalence data is limited to, on average, only 21.5 observations over a period of 31.4 years spanning 1960–2012 [39]. As such, it misses much of the crucial period in the earlier parts of the 20th century during which smoking steadily gained popularity in many countries. However, historical national cigarette consumption data is available for the same 24 OECD countries plus Romania for an average of 78.4 observations over a period of 82.2 years spanning 1900-2012 [40, 41]. Since our model is specified in terms of smoking prevalence, we estimate smoking prevalence from cigarette consumption in order to exploit the much richer cigarette consumption data for model fitting purposes. First, we assume a linear relationship between smoking prevalence *x*(*t*) and smoking consumption *c*(*t*)

(3) $$ x(t) = C c(t) + B.  $$

Next, we calculate estimates $\widehat {C}$ and $\widehat {B}$ by regressing smoking prevalence *x*(*t*) on tobacco consumption *c*(*t*) for all years for which both measurements are available. The results of this regression are summarized in Table A.2 of the Additional file 1, which illustrates that the assumption that *x* and *c* are linearly related does not hold equally well for all countries. In order to restrict ourselves to the cases where the assumption of linearity between *x* and *c* is valid we restrict ourselves to the seven OECD countries with *R*^2^≥0.7, *p*<0.001, and *n*_*obs*_≥15: Australia, Canada, France, New Zealand, Sweden, the United Kingdom, and the United States. We display the raw data for these seven OECD nations in Fig. A.1 of the Additional file 1. The smoking prevalence for these seven OECD countries is then estimated from tobacco consumption using the relationship

(4) $$  \hat{x}(t) = \widehat{C} c(t) + \widehat{B}.  $$

Note that survey-based prevalence data are susceptible to noise stemming from variations in the survey methodology. In particular, prior to performing the linear regression of *x* on *c* for France, we removed the outlier *x*(1960)=0.32 since it is inconsistent with the rest of the data for France, see Fig. A.1(c) of the Additional file 1. Specifically, the Grubbs test on $x / \hat {x}$ indicates that the 1960 data point is a significant outlier (*p*<0.05). This can also be seen intuitively: from *t*=1960 until the next measurement at *t*=1965 smoking prevalence drops from *x*(1960)=0.32 to *x*(1965)=0.25 (a decrease of 21.9 %), while cigarette consumption steadily increases from *c*(1960)=3.6 to *c*(1965)=4.1 (an increase of 13.9 %). Given the population in France in 1960 (45.5 million) and in 1965 (48.6 million) [42], this would correspond to an increase in the average mass of cigarettes smoked (in grams) per smoker per day from 11.3 to 16.4 (an increase of 45.1 %) over a short 5 year period. This is in sharp contrast with the relatively stable relationship between *x* and *c* for France’s remaining data points and justifies the exclusion of the outlier *x*(1960)=0.32. With the outlier removed, France satisfies our data quality requirements for inclusion in the set of seven OECD countries (*R*^2^≥0.7, *p*<0.001, and *n*_*obs*_≥15).

Our assumption of linearity between smoking prevalence *x* and cigarette consumption *c* is not perfect, but it appears to be satisfied at most times in countries where both data sets are available. Quadratic or other higher order terms could be included, but additional unknown parameters would have to be introduced and the limitations of our data set (sparsity, noise) mean that there would be little or no improvement in the model’s fit.

#### Proxy Data *n*(*t*): articles published on the health effects of smoking

We calculate the cumulative number of articles published on the health effects of smoking *n*(*t*) by performing a search of the online research database Scopus for papers with

- tobacco, smok*, or cigar* in the title, and
- death, illness, mortality, risk*, tumour*, tumor*, or cancer in the title, and
- medicine, dentistry, nursing, veterinary, health professions, or multidisciplinary in the subject area, and
- plant*, mosaic, botany, smog, fog, and soot not in the title.

**Fig. 3:**
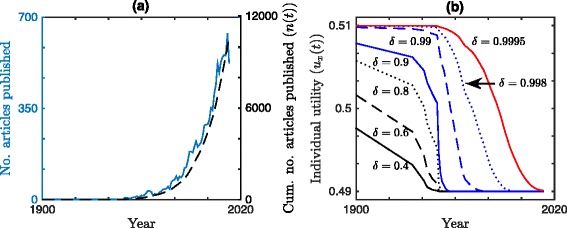
Articles published on the health effects of smoking and the individual utliity function. Articles retrieved by Scopus with search terms (i)-(iv) and individual utility profiles from Eq. (2) for varying values of *δ*. **a** (Left axis - blue, solid) Number of articles published per year and (right axis - black, dashed) cumulative number of articles published *n*(*t*). **b** Discounted utility *u*_*x*_(*t*) from Eq. (2) with *u*_0_=0.51 and *u*_*∞*_=0.49, using cumulative number of articles published *n*(*t*). (Solid black) *δ*=0.4, (dashed black) *δ*=0.6, (dotted black) *δ*=0.8, (solid blue) *δ*=0.9, (dashed blue) *δ*=0.99, (dotted blue) *δ*=0.998, and (solid red) *δ*=0.9995

Items (i)-(iii) are search terms included in order to select for papers researching the health effects of smoking, whereas items (iv) are search terms excluded in order to prevent selection of papers researching the tobacco mosaic virus (plant*, mosaic, botany) and the health effects of atmospheric smoke (smog, fog, soot). This provides us with *n*(*t*) for integer *t*, where time *t* is measured in years. We make the article data available in CSV format in an Additional file 3, which contains three columns: year (*t*), number of articles published in year *t*, and cumulative number of articles published up to and including year *t* (*n*(*t*)). To calculate *n*(*t*) for non-integer and missing values of *t* we use linear interpolation, see Fig. 3(a). Furthermore, Fig. 3(b) displays *u*_*x*_(*t*) from Eq. (2) using *n*(*t*) calculated above for various discount factors *δ* and with *u*_0_=0.51 and *u*_*∞*_=0.49. (For comparison, see Tables 1 and 2 for model-fitted values of *δ*, *u*_0_ and *u*_*∞*_).

**Table 1:** Universal parameters and total error (*E*_2_) resulting from fitting model (1) to the estimated smoking prevalence ($\hat {x}$)

Universal parameters and total error (*E*_2_)		
*b*	*δ*	*E*_2_
1.049	0.9981	0.163

**Table 2:** Local parameters and local error (*E*_*i*,2_ and *E*_*i*,1_) resulting from fitting model (1) to the estimated smoking prevalence ($\hat {x}$)

Country	Local parameters and local error (*E*_*i*,2_ and *E*_*i*,1_)					
(*i*)	*a*_*i*_	*x*_*i*,0_	*u*_*i*,0_	*u*_*i*,*∞*_	*E*_*i*,2_	*E*_*i*,1_
Australia	1.035	0.033	0.551	0.484	0.032	0.015
Canada	1.020	0.083	0.530	0.483	0.020	0.011
France	1.121	0.198	0.543	0.524	0.004	0.005
New Zealand	1.062	0.202	0.525	0.504	0.012	0.010
Sweden	1.076	0.077	0.555	0.503	0.015	0.009
United Kingdom	0.976	0.079	0.513	0.478	0.060	0.018
United States	0.963	0.063	0.513	0.470	0.024	0.013

### Testing the model

#### Phase (i): model fitting/direct test

We fit Eq. (1) to the estimated prevalence, $\hat {x}(t)$. To reduce the dimensionality of the optimization problem, we assume that certain *universal parameters* are constant across countries. Specifically, we assume that *b* and *δ* are universal parameters, and that *x*_*i*_(*t*_*i*,0_)=*x*_*i*,0_, *a*_*i*_, *u*_*i*,0_, and *u*_*i*,*∞*_ are *local parameters* for country *i*, where *t*_*i*,0_ is the first year for which cigarette consumption data (*c*), and hence estimated smoking prevalence data ($\hat {x}$), are available. We denote the smoking prevalence estimated above for country *i* at time *t* by $\hat {x}_{i}(t)$. The time series of estimated smoking prevalences for country *i* is then denoted by the vector $\widehat {X}_{i}$. Analogously, we denote the time series of smoking prevalences predicted by Eq. (1) for country *i* by $\widetilde {X}_{i}$. We solve Eq. (1) using the Matlab differential equation solver *ode45*.

Using the Matlab function *lsqcurvefit* we proceed as follows:

1. Holding universal parameters constant, for each country *i* we find the *x*_*i*,0_, *a*_*i*_, *u*_*i*,0_, and *u*_*i*,*∞*_ that minimize
  $$E_{i,2} = \|\widetilde{X}_{i}-\widehat{X}_{i}\|_{2}^{2}, $$
  where the *L*_2_ norm ∥·∥_2_ for a vector $y=(y_{1},\ldots, y_{n}) \in \mathbb {R}^{n}$ is given by $\|y\|_{2} = \sqrt {\sum _{j=1}^{n} {y_{j}^{2}}}$.
2. Holding local parameters constant for each country *i*, we find the *b* and *δ* that minimize $E_{2} \!= \!\sum _{i}\|\widetilde {X}_{i}-\widehat {X}_{i}\|_{2}^{2} = \sum _{i} E_{i,2}$.
3. Repeat steps (1) and (2) until either
  - the change in the objective function $E_{2}=\sum _{i} E_{i,2}$ is below tolerance *tol*, or
  - the number of iterations exceeds a limit *max*_*itn*_.

We perform the optimization with the initial guess *u*_*i*,0_≡0.51, *u*_*i*,*∞*_≡0.49, $x_{i,0} = \hat {x}_{i}(t_{i,0})$, *a*_*i*_=1, *b*=1, and *δ*=0.9985. We also provide the optimization algorithm *lsqcurvefit* with constraints

$$\begin{aligned} 0&\leq a_{i}, b \leq 2\,\, \text{and}\\ 0&\leq x_{i,0}, u_{i,0}, u_{i,\infty}, \delta \leq 1, \end{aligned} $$

and with parameters *tol*=10^−6^ and *max*_*itn*_=150. The fitting procedure terminates after 114 iterations, the results of which are recorded in Tables 1 and 2 and Fig. A.2 of the Additional file 1. For completeness, Tables 1 and 2 also record the average of the absolute value of the difference between $\tilde {X}_{i}$ and $\hat {X}_{i}$

$$E_{i,1} = \frac{\|\tilde{X}_{i} - \hat{X}_{i}\|_{1}}{\text{length of }\hat{X}_{i}}, $$

where the *L*_1_ norm ∥·∥_1_ for a vector $y=(y_{1},\ldots,y_{n})\in \mathbb {R}^{n}$ is given by $\|y\|_{1}=\sum _{j=1}^{n}|y_{j}|$, and where the length of $\hat {X}_{i}$ is equal to the number of elements of $\hat {X}_{i}$, i.e. the length of $\hat {X}_{i}$ is equal to the number of years for which smoking prevalence estimates $\hat {x}_{i}(t)$ are available. For complete model simulation code with all necessary data files, see Additional file 4.

#### Phase (ii): Test of model implications for *a*

If the model and its interpretation are correct and the balance between individual and social utility is a relevant factor for the temporal dynamics of smoking prevalence, then we expect that the fitted relative conformity parameter *a* will be different for different countries and will capture something meaningful about the individualism/collectivism of a society. To test this we compare with Hofstede’s IDV, an established metric for societal individualism [33] that has been evaluated in most countries. Specifically, by computing the linear regression of *a* on IDV we expect to reveal a significant negative correlation between these two quantities (negative because *a* increases with collectivism while IDV decreases with it).

#### Phase (iii): Test of model implications for slope and peak year

Besides the correlation of *a* with collectivism, we note that another prediction is implicit in model (1). As the relative conformity parameter *a* increases, the model requires that changes in smoking prevalence occur more slowly (this is true for solutions to Eq. (1) for the range of *a* and *u* values corresponding to the observational data). Put another way, societies with higher levels of individualism should experience faster changes in smoking prevalence. Intuitively, when smoking prevalence is low the lack of existing smokers inhibits smoking initiation more strongly in a collectivistic society than in an individualistic society. Thus, we expect the average rate of increase in a collectivistic society to be smaller than in an individualistic society. In contrast, when smoking prevalence is high, and once the deleterious health effects of smoking become widely known and negatively impact individual utility from smoking, the presence of existing smokers inhibits smoking cessation more strongly in a collectivistic society than in an individualistic society. In both cases collectivism acts as a brake on change in the status quo (higher cultural inertia [43, 44]). Specifically, we expect the average slope *s*_*x*_ of the smoking prevalence curves leading up to the peak smoking prevalence increases with Hofstede’s IDV and decreases with *a*, respectively. Here we define the average slope *s*_*x*_ to be

(5) $$  s_{x} = \frac{\hat{x}(t_{max}) - \hat{x}(t_{0})}{t_{max}-t_{0}},  $$

where *t*_0_=1920 is the first year for which smoking prevalence estimates are available in the subset of seven OECD countries, and where *t*_*max*_ is the earliest year for which the maximum tobacco consumption was recorded, see Table A.3 of the Additional file 1.

This reasoning further suggests that the peak year for smoking prevalence *t*_*max*_ should be later in collectivistic societies and earlier in individualistic societies. Specifically, we expect *t*_*max*_ to be significantly negatively correlated with IDV and significantly positively correlated with *a*. Note that our assumption of a linear relationship between national cigarette consumption and smoking prevalence is not needed to establish *t*_*max*_, so the relationship between *t*_*max*_ and IDV is independent of any model assumptions.

## Results

We test the model in three phases, as depicted in Fig. 2. In Phase (i) we calibrate the model using smoking prevalence estimates $\hat {x}_{i}(t)$ derived from the tobacco use data (smoking prevalence and cigarette consumption data). The model predictions about how the relative conformity parameter *a*, the slope *s*_*x*_ and the peak year *t*_*max*_ are related to the level of individualism/collectivism in society are tested in Phases (ii)-(iii) by comparison to an existing measure of individualism/collectivism, i.e. to Hofstede’s IDV. Since the model is calibrated using one set of data (smoking prevalence and cigarette consumption data) and its predictions are verified using a separate data set (Hofstede’s IDV), Phases (i)-(iii) provide significant evidence in support of the model that we developed in Eq. (1).

### Phase (i): Direct test

Figure 1 shows the fit of our model to data sets from the United States and Sweden (additional fits and parameter values are displayed for our set of seven OECD countries in Fig. A.2 of the Additional file 1 and Tables 1 and 2). The average of the absolute value of the difference between smoking prevalence estimates $\hat {x}$ and the output of Eq. (1) ranges from a low of 0.005 for France to a high of 0.018 for the United Kingdom (see *E*_*i*,1_ in Table 2). The good agreement that we found with all data sets provides support for the model.

### Phase (ii): Test of model implications for *a*

**Fig. 4:**
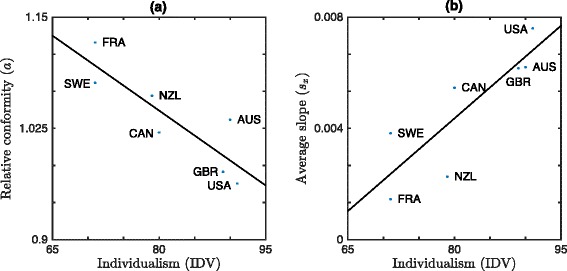
Relative conformity *a* and average slope *s*_*x*_ versus Hofstede’s IDV for seven OECD countries. For both panels the line of best fit is given by a solid line. **a** Relative conformity *a* is negatively correlated with IDV (*ρ*=−0.87, *p*=0.011, best fit slope: −5.6×10^−3^). **b** The positive correlation between average slope in smoking prevalence *s*_*x*_ and IDV (*ρ*=0.85, *p*=0.015, best fit slope: 2.2×10^−4^) is consistent with slower change in collectivistic societies: the average slope is greater in individualistic societies and smaller in collectivistic societies

Panel (a) of Fig. 4 shows the comparison between the fitted *a* values and IDV. As expected, the relative conformity parameter *a* shows significant differences for different countries and is significantly negatively correlated with Hofstede’s IDV (see Table 3). This concordance with independently assessed individualism values supports our model.

**Table 3:** Correlation between IDV, relative conformity *a*, average slope *s*_*x*_, and peak year *t*_*max*_

	7-country subset			25-country set
	*a*	*s*_*x*_	*t*_*max*_	*t*_*max*_
IDV	−0.87(0.011)	0.85 (0.015)	−0.76(0.047)	−0.53(0.006)
*a*	–	−0.92(0.003)	0.88 (0.009)	–

Correlations between IDV, *a*, *s*_*x*_, and *t*_*max*_ are recorded for the seven-country subset. Correlation between IDV and *t*_*max*_ is recorded for the full set of 25 countries. *p*-values are in parentheses. All correlations are significant at the 95% confidence level

### Phase (iii): Test of model implications for slope and peak year

**Fig. 5:**
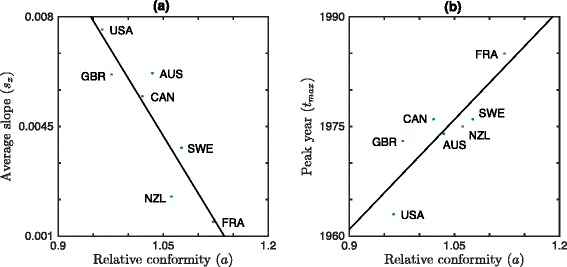
Average slope *s*_*x*_ and peak year of smoking prevalence *t*_*max*_ versus relative conformity parameter *a*. **a** Average slope *s*_*x*_ versus relative conformity parameter *a* (*ρ*=−0.92, *p*=0.003). **b** Peak year *t*_*max*_ versus relative conformity parameter *a* (*ρ*=0.88, *p*=0.009). The line of best fit is given by a solid line

**Fig. 6:**
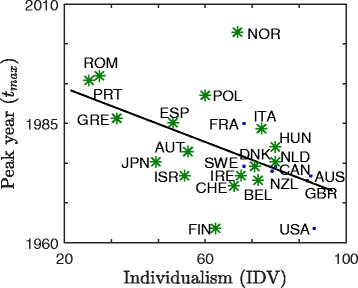
Peak year *t*_*max*_ in cigarette consumption versus Hofstede’s individualism index IDV for 24 OECD countries and Romania. The negative correlation between peak year *t*_*max*_ and IDV in a set of 25 countries (*ρ*=−0.524, *p*=0.008) is consistent with slower change in collectivistic societies: the peak year in tobacco consumption tends to occur later in collectivistic societies and earlier in individualistic societies. The seven OECD countries considered for the mathematical model are indicated by dots (*ρ*=−0.76, *p*=0.047), and the remaining 18 countries are indicated by asterisks. The line of best fit, calculated using all 25 countries, is given by a solid line

Panel (b) of Fig. 4 and panel (a) of Fig. 5 illustrate the relationship between *s*_*x*_ and IDV and between *s*_*x*_ and *a*: that this is indeed the case: the average slope *s*_*x*_ of the smoking prevalence curves leading up to the peak increases with Hofstede’s IDV and decreases with *a*, respectively. Figure 6 illustrates the relationship between *t*_*max*_ and IDV: *t*_*max*_ is significantly negatively correlated with IDV (shown) and significantly positively correlated with *a* (see Fig. 5(b)). Note that our assumption of a linear relationship between national cigarette consumption and smoking prevalence is not needed to establish *t*_*max*_, so Fig. 6 is independent of any model assumptions. All correlations are significant (see Table 3).

We note that fluctuations in the data due to either volatility in tobacco consumption or measurement error may affect reported *t*_*max*_. Smoothing of the data could be applied prior to calculation of peak year, however, the choice of smoothing algorithm is itself arbitrary and unnecessarily complicates our findings without significantly altering the result. For example, consider the seven OECD countries for which we have estimated historical smoking prevalence data $\hat {X}_{i}$. We observe that the model fitting procedure described in the Methods section results in the timeseries $\tilde {X}_{i}$, which we can consider as one possible smoothing of the data $\hat {X}_{i}$. In this case, the measurement for peak year does not change substantially after smoothing for most countries (see Fig. A.2(a)-(e) of the Additional file 1), while the measurement for peak year in the USA would slightly increase from *t*_*max*_=1963 to *t*_*max*_=1967 and the measurement for peak year in the UK would slightly decrease from *t*_*max*_=1973 to *t*_*max*_=1966 (see Fig. A.2(f)-(g) of the Additional file 1). These changes would result in no discernible net change in the relationship between peak year and individualism, but would result in added complexity, and hence, in a greater chance of introducing additional error^1^.

## Discussion

Before discussing the limitations of our model, it is worth discussing the potential effect of confounding variables on our model. Specifically, we argue that the effect of confounding variables on our results are limited, since most potential candidates for confounding variables are actually accounted for implicitly in our modelling framework. Consider, for example, two possible candidates for confounding variables: the wealth (per capita GDP) and the strength of tobacco control policies in each country. Specifically, consider the trend in Fig. 6 where wealthier countries have, on average, earlier peak year in tobacco consumption (*t*_*max*_). This relationship is easily explained by our model, since (a) our model predicts a negative correlation between peak year and Hofstede’s IDV and (b) IDV and wealth (per capita GDP) are highly positively correlated. An alternative explanation for wealthier nations having earlier peak year, however, would be that individuals who are more wealthy are better able to afford cigarettes and, in aggregate, are better able to implement strong anti-tobacco policies: in theory, the former would lead to a more rapid increase in smoking prevalence and the latter would lead to a more rapid decline in smoking prevalence. Although this alternative explanation might seem to be in competition with our model, we argue that it is in fact accounted for implicitly in our modelling framework: both wealth and the strength of tobacco control policies are contributing factors to individual utility from smoking. Furthermore, we note that although the precise timing of anti-tobacco policies is not included in the model, it is reasonable to assume that these initiatives are implemented more frequently and more intensely as the health effects of smoking are better understood - a phenomenon which is modelled using Eq. (2) and proxy data on smoking related publications. In summary, since most potential confounding variables are actually accounted for in, and not in competition with, our modelling framework, the exposure of our results to the effects of confounding variables is limited.

Despite the good match between model predictions and data, a number of limitations remain. First, due to limitations in the quantity and quality of the available smoking prevalence and tobacco consumption data, we are only able to fit the parameters in our model to seven countries, all of which have advanced/developed economies. There is no *a priori* reason to believe that, given adequate sources of data, our model would not generalize to less developed countries with lower income. Indeed, Fig. 6 supports the position that the behaviour in less developed countries is consistent with our mathematical model. Nevertheless, our inability to directly apply our model to a larger set of more diverse countries due to a lack of good data remains a limitation of our work and an area open to future research. Second, we have made an implicit “mean-field” approximation in taking social utility to be a function of the overall smoking prevalence *x*, rather than the local smoking prevalence among contacts in an individual’s social network. Similarly, we have taken individual utility to be uniform across the population (though not in time), whereas a more detailed model might allow for variation with, e.g., gender, age and income. We note, however, that the success of our model in reproducing the trends in smoking prevalence in a manner consistent with its interpretation in the context of individualism/collectivism, despite these limitations, is generally supportive of the modelling framework we have developed. In particular, our results and the data indicate that, when averaging over gender, age and income in a country, a strong net influence remains from societal individualism on the aggregate temporal dynamics of smoking prevalence. Furthermore, if the mechanism in our model did not reflect the reality of the decision-making process for smoking then, even if it still somehow managed to fit the smoking prevalence data, we would not expect to simultaneously find correlation of (a) the relative conformity parameter *a* with Hofstede’s individualism measure IDV (Fig. 4(a)), (b) average slope *s*_*x*_ with IDV (Fig. 4(b)), and (c) peak year *t*_*max*_ with IDV (Fig. 6). Moreover, we would not expect that the sign of these correlations would be consistent with the predictions of the model.

Our findings suggest that the correlation of individualism with faster societal change (as a consequence of a sudden change in personal utility) results from a causative influence as predicted by our model. As already mentioned, other factors such as income levels also correlate with individualism. We certainly cannot exclude that there may be other causative factors. For example, our model in its current form is incapable of explaining differences in smoking prevalence between genders and why these inter-gender differences vary between countries [15, 23]. Nevertheless, we remark that many previously proposed causative factors for differences in observed inter-country smoking dynamics can be accounted for within our modelling framework. In particular, beliefs about the harmful effects of smoking [16], the price of cigarettes [19], socioeconomic status and inequality [17, 18], and government regulation [20–22] have all been cited as potential factors affecting the differences observed in inter-country smoking dynamics. Each of these factors can be interpreted within our modelling framework. For example, beliefs about the harmful effects of smoking, as well as the price of cigarettes, both likely contribute directly to individual utility derived from smoking (*u*_*x*_) and from non-smoking (*u*_*y*_). Moreover, socioeconomic status may affect individual utility from smoking indirectly by affecting an individual’s tolerance for risk and/or how they discount future rewards and costs (i.e. how they discount their future health status) [45]. Addressing the model’s inability to account for gender differences in smoking prevalence and explicitly quantifying the relationship between other causative factors and model parameters in more elaborate models are potential areas for future work.

## Conclusion

Despite the above mentioned limitations, the quantitative mathematical model proposed in this paper, which we derived from basic principles well-documented in the sociology and social psychology literature, appears to match real-world smoking prevalence data from a variety of countries well (to our knowledge, the largest historical data set of this type ever compiled), and all predictions of the model appear to be supported by the data. Indeed, we emphasize the strong support of the model by the data, since the model was calibrated (in Phase (i)) and its predictions were tested (in Phases (ii)-(iii)) using two separate data sets (tobacco use data and Hofstede’s IDV, respectively). In particular, the model predicts that the level of individualism or collectivism of a society may significantly affect the temporal dynamics of smoking prevalence: the strong influence of the personal utility of smoking (and its decrease due to increased awareness of adverse health effects) is predicted to lead to faster adoption and cessation of smoking in individualistic societies than in more collectivistic societies. The significance of this effect can be illustrated by considering the counterfactual scenario of how the smoking prevalence might have evolved in the United States had the United States been less individualistic. Specifically, we estimate that a reduction in the IDV of the United Stated of 2 % would have resulted in a 16 % decrease in the total number of cigarettes smoked between 1920 and 2010 (see Appendix B in Additional file 1 and Matlab code in Additional file 4 for details of this calculation, which makes use of US Census population data included in Additional file 5 [48, 49]).

It has previously been argued that social support mechanisms in collectivistic societies make it more likely that a person will stop smoking [32, 46] based on findings that social support (supportive counselors) can help people to adhere to decisions to quit smoking [14]. In contrast to this behaviour at the individual level, we find that aggregate smoking prevalence decreases more slowly in collectivistic societies. Since the aggregate smoking prevalence is a function of both smoking adoption and cessation, our model suggests that this may be so because social inertia/peer pressure will either inhibit the decision to stop smoking, or encourage the decision to start smoking, more strongly in collectivistic societies than in individualistic societies.

These results suggest that it may be effective to consider culture-dependent strategies for combating the ongoing smoking epidemic. For example, interventions to discourage smoking can be tailored differently in societies or social groups whose cultures differ in how they value individualism versus collectivism [47]. Specifically, consider how the goal of many tobacco control policies is to reduce the individual utility from smoking, often by increasing the cost of cigarettes through sin taxes or by requiring warnings on cigarette packages illustrating the danger of smoking to health. Our results suggest that these tactics will be more successful in individualistic societies and less successful in collectivistic societies. In contrast, tactics that may be more successful in collectivistic societies might focus on social dangers resulting from smoking, for example by emphasizing the association between smoking and low social status [17, 18], or emphasizing the large number of individuals who have already quit. More broadly, these results demonstrate that differences in culture can measurably affect the dynamics of a social spreading process, and that a mathematical model can help to illuminate this phenomenon. We welcome future work comparing a variety of social contagion phenomena across societies. Our model suggests that the increased cultural inertia in collectivistic societies may potentially lead to slower change across a wide spectrum of spreading processes (those where important changes occur in personal utility), a hypothesis that could be supported or rejected by further study.

## Endnote

^1^ Indeed, these changes would increase the statistical significance of our results, but again, we don’t believe that they justify the additional complexity and the introduction of additional arbitrary smoothing parameters.

## Additional files

Additional file 1
**Appendices A and B.** Appendix A: Additional Tables and Figures, Appendix B: Additional Remarks on Model Implications and Study Design. (PDF 380 kb)

Additional file 2
**Smoking prevalence and tobacco consumption data.** CSV file containing four columns: country number as it appears in Table A.1 of the Additional file 1, year (*t*), measurement (*x*(*t*) or *c*(*t*)), and type of measurement (0 indicates a smoking prevalence measurement, while 1 indicates a cigarette consumption measurement). (CSV 35 kb)

Additional file 3
**Proxy data: articles published on the health effects of smoking.** CSV which contains three columns: year (*t*), number of articles published in year *t*, and cumulative number of articles published up to and including year *t* (*n*(*t*)). (CSV 1 kb)

Additional file 4
**Matlab data files and simulation code.** Matlab data files and simulation code used in preparation of this manuscript. (ZIP 21 kb)

Additional file 5
**Proxy data: US population.** CSV containing two columns: year (*t*) and population *N*_*pop*_(*t*). (CSV 1 kb)

## Abbreviations

OECD: Organization for economic co-operation and development
IDV: Hofstede’s individualism index
