# Supplementary material for: The influence of societal individualism on a century of tobacco use: modelling the prevalence of smoking
Source: BMC Public Health. 2015 Dec 22;15:1280. doi: 10.1186/s12889-015-2576-6 (PMC4688941; doi:10.1186/s12889-015-2576-6)
Supplement: Additional file 1 — Appendices A and B. Appendix A: Additional Tables and Figures, Appendix B: Additional Remarks on Model Implications and Study Design. (PDF 380 kb) [file 12889_2015_2576_MOESM1_ESM.pdf]

The influence of societal individualism on a century of tobacco use: modelling the prevalence of smoking
Appendices A and B

J.C. Lang, D.M. Abrams, and H. De Sterck

A Additional Tables and Figures

Table A.1: Summary of data on smoking prevalence x and cigarette consumption c.

			Smoking prevalence (x)		Cigarette consumption per person per day (c)		
No.	Country	Abbrev.	Obs. Period	No. of Obs.	Obs. Period	No. of Obs.	Source
1	Australia	AUS	1964–2010	16	1920–2010	91	[40]
2	Austria	AUT	1972–2006	5	1923–2004	82	[40]
3	Belgium	BEL	1997–2008	4	1921–2011	91	[40]
4	Canada	CAN	1964–2011	29	1920–2010	91	[40]
5	Denmark	DNK	1970–2010	41	1920–2010	91	[40]
6	Finland	FIN	1978–2011	34	1920–2009	90	[40]
7	France	FRA	1960–2010	22	1900–2010	93	[40]
8	Greece	GRE	1998–2009	6	1920–1995	76	[41]
9	Hungary	HUN	1994–2009	4	1920–2012	87	[40]
10	Iceland	ICE	1987–2012	26	1932–1995	64	[41]
11	Ireland	IRE	1973–2007	14	1920–1995	76	[41]
12	Israel	ISR	1996–2010	8	1967–1995	29	[41]
13	Italy	ITA	1980–2012	23	1905–2010	73	[40]
14	Japan	JPN	1965–2011	47	1920–2007	88	[40]
15	Netherlands	NLD	1966–2011	39	1923–1995	67	[41]
16	New Zealand	NZL	1976–2012	28	1920–2009	90	[40]
17	Norway	NOR	1973–2012	40	1927–2011	85	[40]
18	Poland	POL	1996–2009	4	1925–1995	43	[41]
19	Portugal	PRT	1987–2006	4	1940–1995	56	[41]
20	Romania	ROM	–	0	1920–1995	52	[41]
21	Spain	SPA	1985–2011	11	1920–2010	87	[40]
22	Sweden	SWE	1980–2011	32	1920–2006	87	[40]
23	Switzerland	CHE	1992–2007	4	1934–2009	76	[40]
24	United Kingdom	GBR	1960–2010	38	1905–2009	105	[40]
25	United States	USA	1965–2011	36	1920–2010	91	[40]

Table A.2: Result from Eq. (3) regression of smoking prevalence x on cigarette consumption c.

Country	$\widehat{C} \times 10^2$	$\widehat{B} \times 10^2$	R^2	p	n_{obs}
Australia	4.5 ± 1.3	-0.3 ± 8.8	0.80	3.2×10^{-6}	16
Austria	0.0 ± 4.9	24.2 ± 32.4	0.00	0.99	4
Belgium	2.6 ± 20.3	13.0 ± 81.5	0.13	0.64	4
Canada	3.5 ± 0.5	6.3 ± 3.8	0.87	3.0×10^{-13}	28
Denmark	0.0 ± 9.2	40.5 ± 44.4	0.00	0.99	41
Finland	2.0 ± 0.7	15.8 ± 2.8	0.55	1.0×10^{-6}	32
France	1.8 ± 0.5	19.1 ± 2.5	0.72	6.3×10^{-7}	22
Greece	–	–	–	–	0
Hungary	1.9 ± 1.6	17.4 ± 11.2	0.93	3.5×10^{-2}	4
Iceland	4.9 ± 1.2	0.9 ± 7.0	0.93	2.6×10^{-5}	9
Ireland	5.4 ± 1.1	-4.0 ± 7.4	0.93	1.7×10^{-6}	11
Israel	–	–	–	–	–
Italy	4.8 ± 2.5	-0.3 ± 13.2	0.47	6.1×10^{-4}	21
Japan	1.3 ± 3.2	25.7 ± 27.2	0.02	0.43	43
Netherlands	4.8 ± 3.2	20.5 ± 15.0	0.32	4.7×10^{-3}	23
New Zealand	2.0 ± 0.3	18.8 ± 1.4	0.86	2.6×10^{-12}	27
Norway	-7.2 ± 4.3	50.1 ± 10.6	0.24	1.6×10^{-3}	39
Poland	–	–	–	–	0
Portugal	–	–	–	–	1
Romania	–	–	–	–	0
Spain	6.0 ± 6.2	-7.4 ± 41.7	0.38	5.7×10^{-2}	10
Sweden	5.4 ± 0.6	4.3 ± 2.3	0.92	1.7×10^{-15}	27
Switzerland	2.8 ± 5.6	7.2 ± 38.6	0.69	0.17	4
United Kingdom	5.6 ± 0.7	1.6 ± 4.5	0.88	5.3×10^{-18}	37
United States	3.6 ± 0.3	-0.1 ± 2.3	0.95	1.1×10^{-22}	35

$\pm$ indicates 95% confidence intervals. We report R^2 values for the linear regression of x on c, the p-value of the correlation between x and c, and the number of years for which both x and c measurements are available, n_{obs}.

Table A.3: Hofstede's Individualism Index IDV and peak year in cigarette consumption (t_{max})

Country	IDV	Peak year (t_{max})
Australia	90	1974
Austria	55	1979
Belgium	75	1973
Canada	80	1976
Denmark	74	1976
Finland	63	1963
France	71	1985
Greece	35	1986
Hungary	80	1980
Iceland	60	1984
Ireland	70	1974
Israel	54	1974
Italy	76	1984
Japan	46	1977
Netherlands	80	1977
New Zealand	79	1975
Norway	69	2004
Poland	60	1991
Portugal	27	1994
Romania	30	1995
Spain	51	1985
Sweden	71	1976
Switzerland	68	1972
United Kingdom	89	1973
United States	91	1963

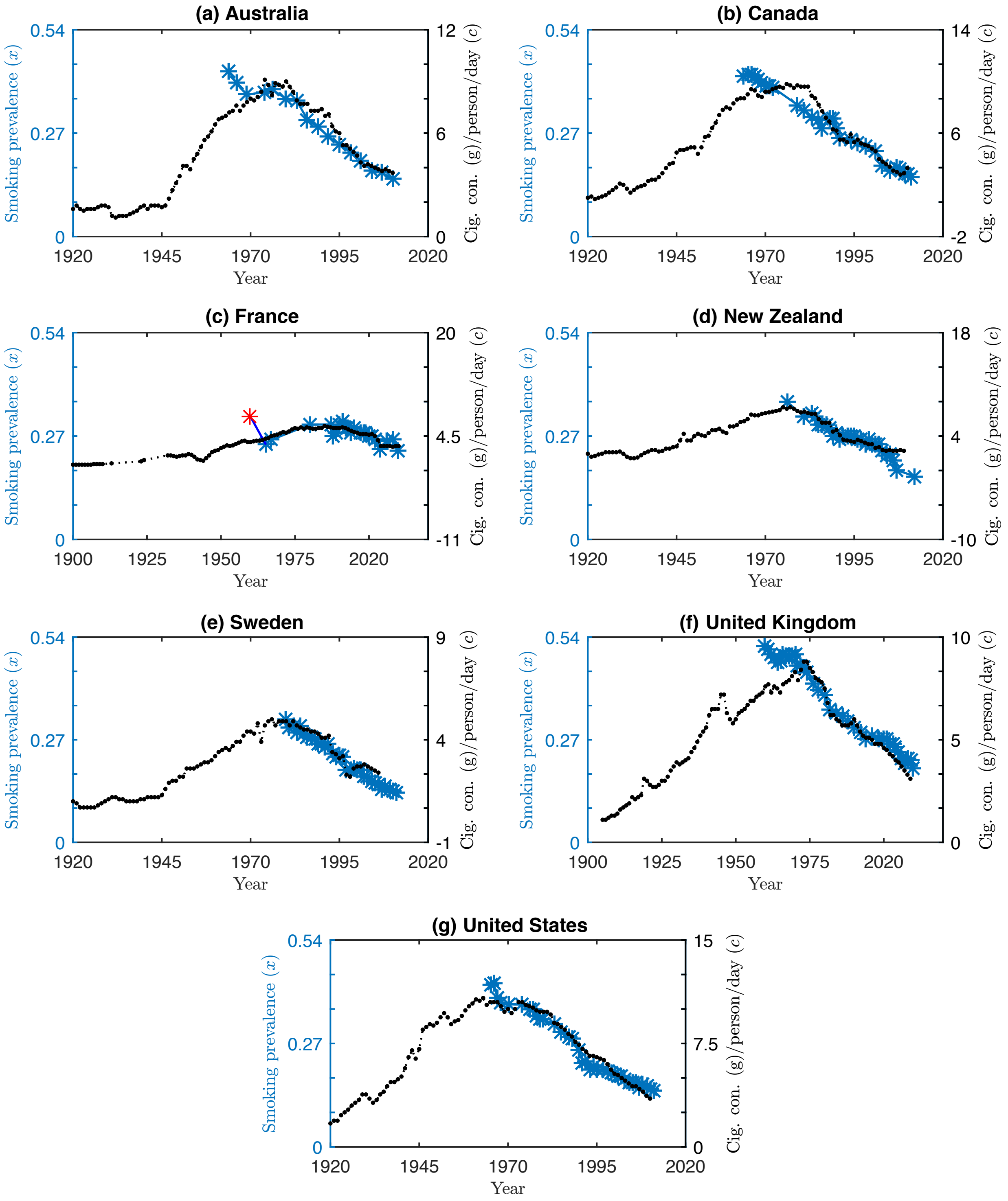

Figure A.1: **Raw smoking prevalence and cigarette consumption data.** Raw smoking prevalence x (blue asterisks, left axis) and raw cigarette consumption c (black dots, right axis) versus time. Raw cigarette consumption data is given in grams per person per day. A single outlier for smoking prevalence (x) for the country of France (panel c) is denoted with a red asterisk.

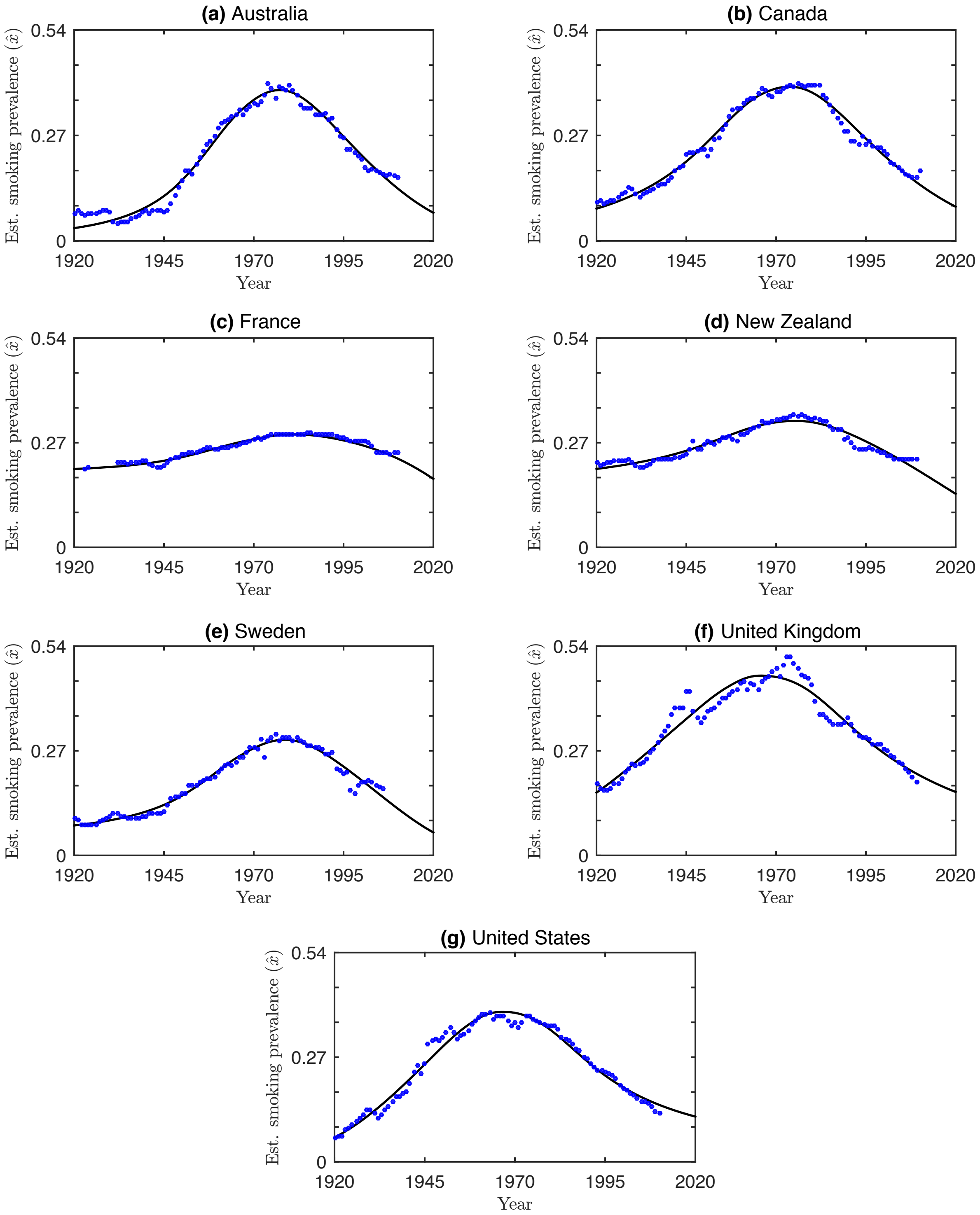

Figure A.2: **The result of fitting Eq. (1) to the estimated smoking prevalence $\hat{x}$.** Estimated smoking prevalence values $\hat{x}$ are given by blue dots.

B Additional Remarks on Model Implications and Study Design

B.1 A Counterfactual Scenario

In this section we illustrate the effect size of individualism/collectivism on the dynamics of the smoking epidemic by considering a simple counterfactual scenario. Specifically, holding all other fitted parameters constant, we consider how the smoking epidemic in the United States might have evolved if the United States (IDV=91 and $a = 0.963$) were about 2% less individualistic (IDV=89 and, using the slope from Fig. 4(a), $a = 0.974$). Fig. A.3 plots an estimate for the number of cigarettes smoked per year (in trillions) versus time. Integrating the difference between the number of cigarettes smoked per year versus time for the United States with fitted ($a = 0.963$, solid line) and counterfactual ($a = 0.974$, dashed line) relative conformity implies that, according to our model, if the United States had 2% lower individualism during the 90 year period from 1920–2010 then there would have been approximately 7×10^{12} fewer cigarettes smoked. This is equivalent to a 16% decrease in the number of cigarettes smoked.

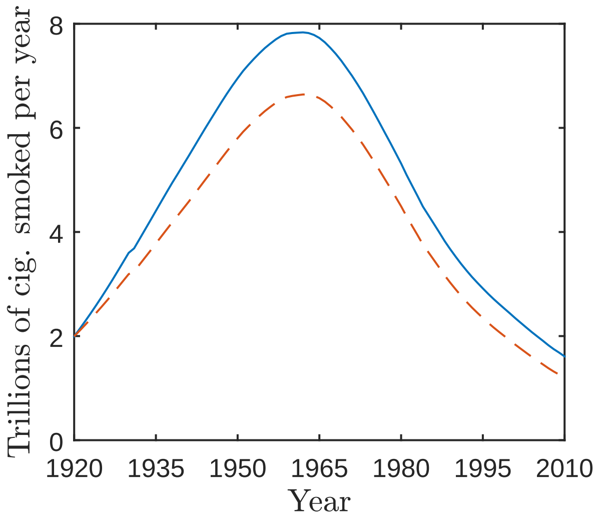

Figure A.3: **Solution to Eq. (1) for the United States with $a = 0.963$ (solid) and $a = 0.974$ (dashed).** Parameters x_0, b, u_0, u_∞, and δ are as reported for the United States in Table 1.

The number of cigarettes smoked per year is estimated as follows. First, we observe that for year t the number of cigarettes smoked per smoker per day is[1] $\mathcal{C}_d(t) = c(t)/x(t)$. Therefore, using Eq. (4) we find that the number of cigarettes smoked per smoker per year $\mathcal{C}_a(t) = 365 \times \mathcal{C}_d(t)$ can be bounded. For example, in the case of the United states, where $\hat{B} < 0$, we find that $\mathcal{C}_a(t)$ is bounded by

$$\begin{aligned} 1.02 \times 10^4 = 365 \times \hat{C}^{-1} \times \frac{365 \text{ days}}{\text{year}} \\ \leq \mathcal{C}_a(t) = \frac{1 - \hat{B}/\hat{x}(t)}{\hat{C}} \times \frac{365 \text{ days}}{\text{year}} \\ \leq \frac{1 - \hat{B}/\min \hat{x}(t)}{\hat{C}} \times \frac{365 \text{ days}}{\text{year}} = 1.04 \times 10^4. \end{aligned}$$

Since the lower and upper bounds are relatively tight, we estimate the number of cigarettes smoked per smoker per year to be the average of the lower and upper bounds

$$\bar{\mathcal{C}}_a \approx \frac{2 - \hat{B}/\min \hat{x}(t)}{2\hat{C}} \times \frac{365 \text{ days}}{\text{year}} \approx 1.0 \times 10^4.$$

[1] Assuming 1.002 cigarettes per gram, as in [39,40].

We cross-check this estimate with the direct estimate of $\bar{\mathcal{C}}_a$ taken by averaging $c(t)/x(t)$ for all times where both measurements are available in the raw data (data shown in Fig. A.1). These two estimates agree to two significant figures. Finally, we estimate the number of cigarettes smoked per year to be

$$\hat{x}(t) \times N_{pop}(t) \times \bar{\mathcal{C}}_a,$$

where $N_{pop}(t)$ is the total population at time t. The total population for the United States is taken from US census estimates [47,48] and is given CSV format in the additional file, *Additional File 4.csv*, which contains two columns: year (t) and population $N_{pop}(t)$.

We emphasize that in the counterfactual scenario described above we have only changed a for the United States while keeping all other fitted parameters constant, merely to illustrate that the effect of small changes in a in the model can be large. Therefore, the broad variation in the fitted a across countries, as illustrated in Fig. 4(a), can indeed be expected to lead to a large effect size on cigarette consumption. Note, however, that the results from this counterfactual scenario do not imply that less individualism automatically means lower cigarette consumption, since countries with lower IDV (higher a) than the United States also tend to differ for other fitted parameters and quantities in the model, resulting in substantially different solutions to Eq. (1).

B.2 Order of Model Development and Additional Analyses

The mathematical model was proposed and developed before the data sets were compiled. Following the specification of the model no modifications were made or required to produce the reported results. The correlation between a and IDV was investigated after fitting the model to the data, and strong negative correlation was obtained as a confirmation of the mechanism proposed in the model. In a subsequent step, to further corroborate the hypothesis that societal individualism influences the temporal dynamics of smoking prevalence at the population level, the correlation between IDV and t_{max} was also confirmed for the raw smoking data, independent of the mathematical model. No analysis was performed with additional variables. However, the sensitivity of the model to several assumptions was tested. For example, and as already mentioned, one alternative to the discounting function presented in Eq. (2) was tested: we assumed a step-function individual utility function that took value u_0 for $t_0 \leq t^*$ and u_∞ for $t > t^*$. We also tested the model for various combinations of local and global parameters with both utility functions. For example, whereas in our model δ and b were taken to be global parameters and $x_{i,0}$, $u_{i,0}$, $u_{i,\infty}$, and a_i were taken to be local parameters, we also tested the cases where (a) b was the only global parameter and $x_{i,0}$, $u_{i,0}$, $u_{i,\infty}$, a_i, and δ_i were taken to be local parameters, (b) a and b were taken to be global parameters and $x_{i,0}$, $u_{i,0}$, $u_{i,\infty}$, and δ_i were taken to be local parameters, and (c) a, b, and δ were taken to be global parameters and $x_{i,0}$, $u_{i,0}$, and $u_{i,\infty}$ were taken to be local parameters. These variations confirmed that our modelling procedure was robust, i.e. these variations all produced qualitatively similar results.
